# Assessing the interruption of the transmission of human helminths with mass drug administration alone: optimizing the design of cluster randomized trials

**DOI:** 10.1186/s13071-017-1979-x

**Published:** 2017-02-17

**Authors:** Roy Anderson, Sam Farrell, Hugo Turner, Judd Walson, Christl A. Donnelly, James Truscott

**Affiliations:** 1 Department of Infectious Disease Epidemiology, London Centre for Neglected Tropical Disease Research, School of Public Health, Imperial College London, St Mary’s Campus, London, W2 1PG UK; 2 DeWorm3, Natural History Museum London, London, UK; 3 Departments of Global Health, Medicine, Pediatrics and Epidemiology, University of Washington, Seattle, USA

**Keywords:** Soil-transmitted helminths, Interrupting transmission, Cluster randomized trial design, Stochastic models of transmission, Mass drug administration impact

## Abstract

**Background:**

A method is outlined for the use of an individual-based stochastic model of parasite transmission dynamics to assess different designs for a cluster randomized trial in which mass drug administration (MDA) is employed in attempts to eliminate the transmission of soil-transmitted helminths (STH) in defined geographic locations. The hypothesis to be tested is: Can MDA alone interrupt the transmission of STH species in defined settings? Clustering is at a village level and the choice of clusters of villages is stratified by transmission intensity (low, medium and high) and parasite species mix (either *Ascaris*, *Trichuris* or hookworm dominant).

**Results:**

The methodological approach first uses an age-structured deterministic model to predict the MDA coverage required for treating pre-school aged children (Pre-SAC), school aged children (SAC) and adults (Adults) to eliminate transmission (crossing the breakpoint in transmission created by sexual mating in dioecious helminths) with 3 rounds of annual MDA. Stochastic individual-based models are then used to calculate the positive and negative predictive values (PPV and NPV, respectively, for observing elimination or the bounce back of infection) for a defined prevalence of infection 2 years post the cessation of MDA. For the arm only involving the treatment of Pre-SAC and SAC, the failure rate is predicted to be very high (particularly for hookworm-infected villages) unless transmission intensity is very low (R_0_, or the effective reproductive number R, just above unity in value).

**Conclusions:**

The calculations are designed to consider various trial arms and stratifications; namely, community-based treatment and Pre-SAC and SAC only treatment (the two arms of the trial), different STH transmission settings of low, medium and high, and different STH species mixes. Results are considered in the light of the complications introduced by the choice of statistic to define success or failure, varying adherence to treatment, migration and parameter uncertainty.

## Background

The past decade has seen much expansion in mass drug administration (MDA) programmes to control helminth infections in regions with endemic infection. The major helminths, in terms of the global burden of disease, are *Wucheria bancrofti* (lymphatic filariasis - LF), *Onchocerca volvulus* (Onchocerciasis), *Schistosoma* spp. (schistosomiasis - SCH) and the soil-transmitted helminths (STH - *Ascaris lumbricoides*, *Trichuris trichuria* and the hookworms, *Necator americanus* and *Ancylostoma americanus*). For LF and onchocerciasis, MDA is community based, covering all age groups. Progress in many countries with endemic LF and onchocerciasis infection has been very good, with high coverage achieved and concomitantly, prevalence has fallen to very low levels. For the schistosomes and STH, MDA is typically targeted at pre-school aged children (Pre-SAC) and school aged children (SAC), although in areas with LF control programmes the use of community wide MDA with albendazole as part of dual therapy for LF also acts to control STH. The objective of the World Health Organisation (WHO) for STH is morbidity control in those age groups viewed as most at risk.

Coverage of STH treatment has risen slowly over the past decade with figures running just above 63.3% for SAC of those judged to need treatment and around 48.2% for Pre-SAC coverage in 2015 [1]. The target for 2020 is 75% for both Pre-SAC and SAC and it is hoped that the steady increase can be maintained to meet this target [2, 3]. The picture for the schistosomes is less encouraging, SAC and Adult coverage being around 42.2 and 11.7%, respectively.

In recent years, discussion has turned to the question of whether the emphasis of the WHO strategy for both STH and SCH should shift from morbidity control to the interruption of transmission [4, 5]. One reason for this‚ is a growing body of analysis that suggests that targeting Pre-SAC and SAC alone is unlikely to stop the transmission of STH in most settings, due to a large reservoir of infection in the adult age classes. This is especially true for hookworm, where the majority of worms are typically harboured by adults [6, 7]. In very low transmission settings just treating children could in principle interrupt transmission, but this is only true for very low values of the basic reproductive number, R_0_, which is an overall measure of transmission success in a defined location in the absence of any density-dependent constraints [8]. Throughout this paper, we use the magnitude of R_0_ to characterise the intensity of transmission in a defined setting, as is conventional in much of infectious disease epidemiology but less so at present in helminthology. For dioecious macroparasites, R_0_ defines the average number of offspring produced by a female worm that survive to reach reproductive maturity in the human host. If this number is less than unity in value in a defined location, the parasite cannot persist. The aim of control is therefore to reduce R_0_ < 1. In the presence of density-dependent processes, such as a reduction in mating success in dioecious species when worm burdens are low, transmission can fall below the level required to maintain the parasite within the human host population even when the value of R_0_ is a little above unity in value [8].

An obvious corollary of the maintenance of a reservoir of infection in adults, is that MDA must continue forever in the absence of improvements in the prevailing sanitation and water supply provisions in resource poor settings where STH infections are endemic. Improvements in sanitation and hygiene, that act to reduce egg or larval contamination of human habitats, act to reduce the value of the intrinsic value of R_0_, provided they are sustained indefinitely.

If emphasis shifts to transmission interruption, the question emerges as to whether or not transmission can be interrupted by MDA alone, acknowledging the practical difficulties of improving sanitation and hygiene to a level that can provide benefit in many settings. A series of recent analyses, based on deterministic and stochastic models of helminth transmission and MDA, suggest that interruption of transmission may be possible using MDA alone, even in high transmission settings, provided drug coverage is high and all age groups in the community are targeted [3, 6, 9].

The use of mathematical models in neglected tropical disease (NTD) research is relatively recent, in contrast to other areas of infectious disease epidemiology. As such, model predictions are not yet an integral part of policy formulation. This is despite the fact that the theory underpinning recently published mathematical models of the transmission dynamics of helminths and the impact of MDA dates back to the mid-1980s [10–12]. In addition, the dynamics of helminths are in general much more predictable than many of the directly transmitted viral and bacterial infections due to the apparent absence of significant acquired immunity such that post-treatment infection levels bounce back to pre-control levels in a monotonic manner. This stability is in part due to the relatively long life expectancy of the adult worms in the human host (1 to 10+ years depending on the species of helminth).

Evidence to support changes in WHO policy for control or elimination of STH by MDA should be based on the highest quality evidence available, ideally from randomized trials designed to test the hypothesis derived from theory that MDA alone can interrupt the local transmission of STH. Under a programme funded by the Bill and Melinda Gates Foundation, entitled DeWorm3, such trials are being planned to start in mid 2017. At the centre of the design of these trials is the question ‘How do you measure transmission interruption?’ This question is the topic of this paper. It is addressed at two levels. First, we examine what is meant by the phrase ‘transmission interruption’ for a dioecious helminth with long-lived life-cycle stages. We then turn to the practical issue of how do you determine if you have been successful, given the stochastic nature of the real world? Heterogeneities in trial settings intrude very directly on trial design, sample size choice and the likely probability distributions of different outcomes. To address this uncertainty, stochastic models are employed which give a likelihood of any given outcome by defining a probability of observing the outcome.

## Methods

### Interruption or elimination of parasite transmission

The word ‘control’ has many connotations in the field of public health. For infectious diseases‚ WHO uses the 1998 definitions proposed by Dowdle [13]. He defined control as a reduction in the incidence, prevalence, morbidity or mortality of an infectious disease to a locally acceptable level; elimination as reduction to zero of the incidence of disease or infection in a defined geographical area; and eradication as permanent reduction to zero of the worldwide incidence of infection [13].

His definitions were largely framed for microparasitic infections, where prevalence is the major epidemiological measure and incidence is defined as new infections (change from susceptible to infected) per unit of time. For the macroparasitic helminths, where parasite burden determines morbidity, incidence can be defined either as the acquisition of new individual parasites which may or may not influence prevalence depending on the worm load in the exposed host, or by the conversion of an uninfected person to an infected individual. The definitions of Dowdle still hold with some minor modification.

The elimination of transmission is the reduction of the establishment of new adult parasite infections in the human host to zero in a defined locality. This state will arise over many years, in which the rate of establishment slowly decays to zero after many rounds of mass drug administration. However, some important biological concepts underpin this state. The first is central to the study of the epidemiology and control of infectious diseases and concerns the basic reproductive number, R_0_, which must exceed unity in value for parasite persistence [12].

However, for dioecious species, such as all of the nematode parasites of humans, this concept is modified by the need for a female worm to find a male mate in the host, given that the adult worm cannot leave the host to complete this task. In other words, in the case of STH species, both male and female worms must be in the same host to produce fertile offspring which exit the host as eggs or larvae and perpetuate the life-cycle. As such a critical mean adult parasite density exists in the host population to sustain effective sexual reproduction. This was first noted by Macdonald [14] for schistosome species, and elaborated on by May [15], to include the assumption that worms are aggregated in their distribution in human communities. Macdonald had assumed the distribution was Poisson but many observational epidemiological studies subsequently demonstrated that the distributions are highly aggregated and negative binomial in form with aggregation parameter k which varies inversely with the degree of worm clumping [10, 12]. The studies of Macdonald [14] and May [5] led to the concept of a transmission breakpoint in the mean worm load, below which the attractor of the dynamical system is parasite extinction. The theory behind this concept is laid out in Anderson & May [12], and is based on a system of differential or partial differential equations describing the population dynamics of the adult parasite that have three equilibrium points for the mean worm burden per host in a defined human community; namely, two stable points which are endemic parasite infection and parasite extinction, separated by an unstable point which is the ‘transmission breakpoint’. The magnitude of this breakpoint is strongly influenced by the degree of worm aggregation in the human host. High aggregation increases the likelihood of the parasite finding a mate and therefore lowers the value of the breakpoint mean worm load. A further biological factor influences the value of the breakpoint, namely, the sexual habits of the parasite. Parasites may be monogamous or polygamous, although little is understood about these proclivities for the helminth parasites of humans. It is widely assumed that STH species are polygamous and schistosomes are monogamous, although the hard evidence for this is very limited at present. The transmission breakpoint is lower when parasites are polygamous, since a single male can successfully mate many females in highly aggregated populations when males are scarce. It is important to note that the breakpoint may be crossed even when R_0_ is slightly greater than unity in value, since the definition of this parameter is based on population growth in the absence of density-dependent constraints. Mating success is a density-dependent process, since its magnitude decreases as worm burdens fall [8].

The net outcomes of the existence of a transmission breakpoint or threshold are threefold. First, and most importantly, if this breakpoint is crossed say by the action of MDA, the extinction state is an attractor which, once achieved, is stable in the absence of parasite (and hence host) immigration. Second, its existence implies that the critical point for parasite extinction (and hence the interruption of transmission) is not R_0_ < 1, but a value of R_0_ which results in the mean worm load falling below the breakpoint. This may be a value significantly above unity, such as a value of 2 to 3, depending on the probability distribution of parasite numbers per host and sexual habits (monogamous or polygamous). The third is less obvious. Once the breakpoint threshold is crossed (defined by either a mean worm burden perhaps measured indirectly by egg output in faeces, or by the prevalence of infection) by a defined MDA coverage - the attractor is parasite extinction. Even if MDA stops with infection still present, the parasite population will move to extinction (without any immigration of new infections), but as noted above, it will take time to achieve ‘no new infections’. The epidemiological measurement of the breakpoint is based on the probability distribution of parasite numbers per host and either of the two summary statistics of this distribution can be employed; namely, the mean, or the proportion infected which defines the prevalence of infection. Note, however, the relationship between these two summary statistics is critically dependent on the degree of worm aggregation. For example, in the case of a negative binomial distribution, which is commonly observed for STH (based on worm expulsion or egg output), the relationship where M is the mean, P is the proportion infected and k is the aggregation parameter, is given by

1 $$ \mathrm{P}=1-{\left(1+\mathrm{M}/\mathrm{k}\right)}^{-\mathrm{k}} $$

In practice, within STH parasite control programmes, many complications surround these concepts including the definition of the spatial region within which elimination is targeted (spatial scale), the sensitivity of diagnostic tools to determine the prevalence and intensity of infection, immigration and emigration of people to and from the defined area, the demography of the host population, and control programme impact on the degree of parasite aggregation in the communities targeted (changing values of k in Eq(1)).

Before turning to the description of an approach to randomized trial design to test the concept of transmission elimination, some issues surrounding what must be measured are briefly addressed.

### Migration and demography

**Fig. 1:**
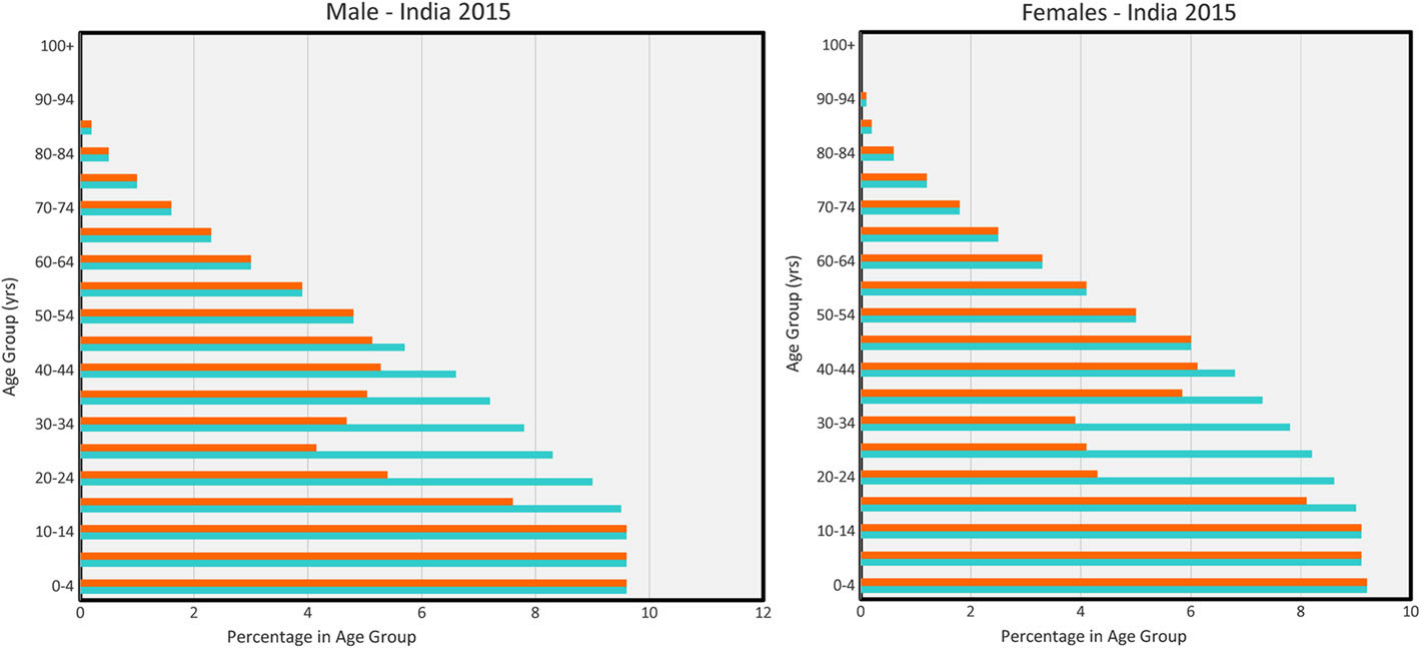
True demography and sampled demography. Demography of India 2015 (source http://www.census.gov/population/international/data/idb/informationGateway.php) (*blue* bars are the full population; *orange* bars are the sampled population)

In regions where STH are endemic, poverty and concomitantly poor employment opportunities are widespread. As a direct consequence, migration in and out of communities, typically by young adult males, to find employment is common. Figure 1 shows an illustrative example for the age and gender structured demography of a community in India in 2015 (data from the US Bureau of Population and Census; http://www.census.gov/population/international) and a hypothetical distribution of those in the community who are sampled for STH infection by their presence or eligibility. For males, many adults are absent, while for females those in the ‘pregnancy’ age classes are not eligible for treatment with albendazole or mebendazole due to specifications in the WHO guidelines for the treatment of pregnant women. This illustrative example shows clearly that treatment coverage calculations designed to move the mean worm burden below the transmission breakpoint must take account of the ‘true’ demography and the ‘sampled/treated’ population. Second, it also illustrates the potential dangers of migrant labour returning to the communities and reintroducing infection if they have not been treated when absent or not immediately on their return. Trials should be designed to take account of these factors.

### The importance of measuring adherence to treatment

A recent review by Shuford and colleagues [16]‚ of adherence to drug treatment in MDA control programmes for helminth infections, revealed that very few studies had recorded both coverage as a fraction of the target populations, and the influence of age and gender on adherence. Accurate data on adherence, with appropriate stratifications, are essential as is a longitudinal component to adherence estimates for individuals. For example, published studies to date do not record the behaviour of individuals over multiple rounds of treatment. Clearly, if there are persistent non-adherers to treatment they will act as a reservoir of infection even if overall coverage is high. In trial designs to test transmission interruption, longitudinal adherence of individuals should be carefully recorded, and where possible directly observed methodologies should be employed.

### Diagnostics

**Fig. 2:**
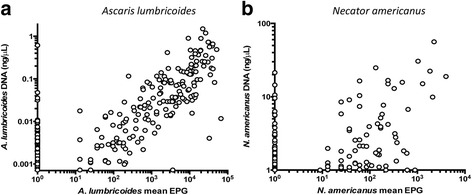
qPRC compared with Kato Katz. qPCR diagnostics test results for **a** *Ascaris lumbricoides* and **b** *Necator americanus* compared with eggs per gram of faeces determined by Kato Katz (from [17])

New diagnostic tools have recently been developed for STH infections to replace the gold standard eggs per gram of faeces measures such as Kato Katz. One example is qPCR (quantitative polymerase chain reaction) which has been tested as a quantitative tool for both egg output in stools and worm burdens by Easton and colleagues [17]. Such studies reveal that at low infection intensity levels, qPCR is much more sensitive in detecting the presence of eggs in stools reflecting low worm burdens. Clearly, in elimination of transmission studies, highly sensitive quantitative tests are ideally required that are well calibrated against existing diagnostic tools such as Kato Katz. An example is presented in Fig. 2 from the work of Easton and colleagues [16]. It records a reasonably tight linear relationship (on a log-log scale, with values define as log(KK + 1)) between the concentration of egg antigen DNA in faecal samples (ng/μl) and measures of eggs per gram of faeces by Kato Katz (KK). Note how many qPCR tests are positive for zero Kato Katz results. In randomized trials, especially as the transmission threshold is approached after multiple rounds of MDA at high treatment coverage, qPCR tests are clearly to be preferred. In country-wide MDA programmes, the costs of such tests and the associated equipment plus labour implications are yet to be evaluated [17–19].

### Species composition and drug efficacy

The three most prevalent STH are *Ascaris lumbricoides*, *Trichuris trichuria*, and hookworms both *Necator americanus* and *Ancylostoma duodenale.* The widely used drugs for the treatment of these infections, albendazole, mebendazole and pyrantel pamoate, have differing efficacies against the same species and different species, as reviewed by Vercruysse et al. [20], Levecke et al. [22] and Keiser & Utzinger [21]. For example, with albendazole, reported average efficacies against *Ascaris*, *Trichuris* and hookworm are 99, 50 and 95%, respectively. In the case of *Trichuris* dual therapy is required using albendazole and ivermectin to gain acceptable levels of efficacy [23]. The effective rate of treatment in MDA programmes is coverage times drug efficacy, and thus both variables should be taken into account in the design of trials.

### Stochastic effects

The real world is replete with heterogeneities, which may or may not be measurable in given transmission and control settings. Chance effects are very important. The use of the word chance is an economy of thought, in the sense it captures what is poorly understood or cannot be measured. In such settings, stochastic models based on the known processes that determine the transmission dynamics of the parasites are required to assess the probability distribution of possible outcomes such as success in breaking transmission. Typically, the mean of this stochastic distribution will be equivalent to the single outcome predicted by a deterministic model. However, this is not always true if many nonlinearities are present in the biological system under study, as may be the case for the transmission dynamics of helminths with sexual reproduction and density-dependence in key processes such as egg production [12, 24, 25]. In randomized trials of MDA impact on helminth transmission, stochastic effects will be very important and hence must be accounted for in trial design. A further important advantage of individual-based stochastic models is their ability to present individual patterns of adherence to treatment, and predisposition to light or heavy infection, over time.

### Simulation of randomized trials

Clinical trials are costly to perform to test a new drug therapy, or a community-based approach to disease control using vaccines or drugs. Increasingly, the pharmaceutical industry is turning to simulation techniques to assess the likely impact of a given trial design and defined endpoints to help reduced costs and increase precision before implementation [26]. Computer simulation of randomized trials, sometimes based on well-defined mathematical models of the key biological and epidemiological processes, has evolved over the past two decades from a simple instructive game to detailed simulation models yielding pharmacological and disease outcome results [27].

Such an approach has rarely been used in community-based randomized trials for NTDs and not at all for the STHs. Given the challenge of accurately measuring transmission interruption in heterogeneous settings, simulation techniques are suggested in the following section which describes a potential method for the design of a randomized cluster-based trial for helminth transmission interruption, using both community wide and Pre-SAC- plus SAC-based MDA treatment programmes.

### A methodological approach to trial design based on the transmission dynamics of STH under repeated rounds of MDA

Before starting to develop a trial design the outcome (transmission elimination or failure to eliminate) that is been tested needs clear definition, as does the statistic to be used to measuring this outcome. This involves all sorts of variables such as, for example, diagnosis specificity and sensitivity of the infection/no-infection status of a participant. We consider a series of issues on which decisions are required in the following subsections.

The first task in trial design is to assess which community-based approach is to be adopted in terms of which age groups are to be targeted, what level of drug coverage in each age group is required to break transmission, how frequently should it be delivered, what transmission settings should the trial be conducted in (low, medium or high transmission settings or all three), over what duration of time is the trial to be conducted, and how long will post-trial monitoring be continued to assess if transmission has ceased once MDA is stopped. The simplest way to do this at the start of design is to be guided by deterministic models, to get some idea of what level of coverage is required, and for how long, to cross the breakpoint, before moving to stochastic formulations to predict the likelihood of the outcomes of elimination and bounce back at various times post the cessation of MDA. It is also important to note that the linkage of the DeWorm3 (http://www.nhm.ac.uk/our-science/our-work/sustainability/deworm3.html) programme of research on STH, to current LF control programmes where community based MDA has often significantly reduced the burden of STH, implies that starting at low to medium levels of STH infection (an effective reproductive number R < R_0_) is the most likely situation in which the trials will be conducted. In these circumstances, a back calculation of the original R_0_ will have to be made based on knowledge (even if partial) of past MDA coverage. The methods required to perform these calculations are described in a companion paper [28].

As mentioned earlier, the purpose of the trial is to ascertain if intensified treatment regimens across defined age classes can interrupt transmission for STHs. Once MDA stops, which age profiles of worm burdens in the host population lead to elimination in the long term and which to parasite bounce back are not known in advance. The main role of stochastic disease transmission models is to try and resolve this issue and to determine what time point post the cessation of treatment would maximise the likelihood of detecting which outcome will emerge in a given village, or village cluster. We need to find a threshold reflecting the parasite burden or prevalence of infection in the population at the end of the study that can discriminate between the two possible long-term outcomes - elimination and bounce back. It needs to be sensitive, specific and easily measurable in the field. The models must also provide clear guidance on when to measure this statistic post MDA cessation to maximize the likelihood of detecting either interruption or bounce back given the non-linear dynamics of the system under MDA and when it ceases. The approach adopted in World Health Organisation guidelines for the filarial worms is to define a prevalence of infection below which (< 1%) MDA can be stopped, since it is assumed that this marks the cessation of transmission. Our approach is different, since the judgement on whether or not transmission has ceased is based on mathematical models of transmission using parameter estimates based on field epidemiological research, not guesswork. In both approaches, however, the quality of the diagnostic measure employed to determine prevalence is clearly very important.

A second important aspect of the trial is that the ability of a given program of treatment to bring about elimination will depend strongly on a number of covariates, some of which can or should be recorded at baseline. Key among these factors are transmission intensity (as represented by prevalence and the mean eggs per gram of faeces (epg) intensity of infection measure, stratified by age group), who has been treated in the past stratified by age group, drug coverage by age group of past treatment, STH species mix and past history of LF treatment. A key feature of the trial will be to investigate how these baseline covariates affect the ability of a given treatment program to bring about elimination. A further desirable property of the elimination statistic discussed above is that its critical value should be, as far as possible, independent of the baseline covariates or, at least, depend on them in a simple predictable way. For the transmission model, different baseline states, such as low medium or high baseline prevalences of infection, can be reproduced through the choice of model parameter values. Hence from the modelling point of view, it is important that the elimination statistic should be largely independent of the parameters that govern the covariates. A more detailed discussion of the dependence of this statistic on various factors in the trial design (number of units, e.g. villages) per cluster, number of clusters and sample size (number of people sampled) per cluster unit, are described in a companion paper [29].

Overall, the model simulations are designed to give clear guidance on cluster randomized trial design once parameter estimates are made from the baseline epidemiology. The key issues can be summarised as follows. (i) What measure of infection to use - prevalence or average intensity? (ii) Which age group or groups should be monitored or should it be the whole population (this depends on the dominant STH species)? (iii) What period of time should elapse post MDA cessation to judge outcome (bounce back or elimination)? (iv) What should be the sample size of people examined in each village and what age groups should they be drawn from? (v) How many villages should be included in a cluster? (vi) Should cluster be chosen on the basis of similarities or proximity (i.e. similar baseline prevalences of infection or spatial colocation)? (vii) How many clusters should be employed? (viii) What should the minimum population size be in a village to be included in a cluster?

#### Arms of the trial and stratifications

DeWorm3 has specified that the trial of breaking transmission will be a cluster randomized design or some variant of this [5]. The concept is simply a development on the framework of the classic randomized controlled trial in which groups of subjects (as opposed to individual subjects) are randomized. Cluster-randomized controlled trials are also known as cluster-randomized trials, group-randomized trials and place-randomized trials. These groups of people may belong to a particular village or community, be in a certain location, or belong to some defined grouping such as an age class. Stratifications in each arm involve the covariates mentioned earlier such as STH species mix, MDA coverage, past history of community-based LF treatment (in other words the baseline prevalence of STH infection), and the prevailing transmission intensity.

What age groups, of the three major groupings Pre-SAC (1–4 years of age), SAC (5–14 years of age) and Adults (15+ years of age), should be targeted for treatment? Given that current WHO policy is to treat only Pre-SAC and SAC, yet analyses point clearly to the merits of treating the whole community if practically feasible [8], one possible approach is for one arm to follow the current WHO policy, and the other arm structured to treat all age groups (bar those less than 1 year old where treatment guidelines suggest no treatment at present for safety reasons) to provide evidence either in support of current policy or for a change in policy to community wide treatment. The broad structure is therefore for only two arms. This is the design laid out for an ongoing study in Kenya [5].

It should be noted that in areas where hookworm is the dominant infection, the arm treating only Pre-SAC and SAC will fail to break transmission since most of the worms are harboured by adults. As published analyses suggest, even for *Ascaris* (treating with albendazole or mebendazle only) and *Trichuris* (treating with albendazole and ivermectin), when transmission intensity is very high (R_0_ values in excess of 3), only treating Pre-SAC and SAC will struggle to break transmission unless previous LF MDA has taken the value of the effective reproductive number, R, to close to unity [4, 6, 8, 30–34].

Ideally, only medium and low transmission settings (overall R_0_ in range 1–2.5, or effective R just above 1 in areas that have experienced significant levels of treatment perhaps due to an ongoing LF control programme prior to the initiation of the trial) should be chosen if the trials are to be completed within 5 years [9]. This would add two stratifications on top of the two age grouping arms to be targeted for treatment. Estimation of R_0_ or the effective R is clearly essential before the trial starts in each village community (a cluster) in order to calculate what level of treatment is required to interrupt transmission. This can be achieved from baseline cross sectional age intensity and age prevalence epidemiological data [28, 35] plus knowledge of past MDA coverage for LF control, ideally stratified by age group.

The next high-level stratification within an arm concerns the STH species mix present in a trial site. If *Trichuris* is the dominant infection, dual therapy may be required. If not, single albendazole (preferable for hookworm) or mebendazole treatment will suffice. Different calculations for the design of the trial will be required depending on what species mix is present in a chosen location.

The final stratification concerns the frequency of treatment. More frequent treatment, such as every 6 months, can speed the movement towards transmission interruption, especially in areas of high transmission for *Ascaris* and *Trichuris*. Weighed against this, however, is the logistical framework required to deliver very frequent treatment at scale, and the disruption to the communities in which treatment is delivered. For these reasons the calculations that follow are based on annual treatment.

#### Deterministic calculations of breakpoint surfaces

Calculations of the breakpoints in transmission, in terms of the mean worm burden or prevalence of infection below which transmission is interrupted, have been published over the past few years for each of the major STH species and schistosome species [4, 6] using different levels of coverage for the three major age groupings Pre-SAC, SAC and Adults [4, 6, 9, 33–35]. For ethical reasons, all individuals who are diagnosed with infection at any time point and in any arm of the trial must be treated.

In all such calculations, the duration of time required to cross the breakpoint is a key concern. Ideally within a 5-year horizon, 3 years would be taken up with three annual rounds of treatment and 2 years of follow up to assess if transmission is eliminated. The models on which these developments have taken place are those described by Anderson [10], Anderson & May [11, 25] and Anderson & Medley [31].

An illustration of the time predicted to be required (given annual treatment) to cross the breakpoint, for various levels of effective treatment coverage in Pre-SAC, SAC and Adults, is presented in Table 1 for hookworm and *Ascaris*, both in medium intensity transmission settings. The vertical axis denotes coverage of Pre-SAC and SAC combined, while the horizontal axis denotes coverage of adults. The figures within the Table 1 are the number of years predicted to be needed, at the defined coverage levels, to break transmission; ‘na’ denotes that the defined levels of coverage will not break transmission. Note that for most combinations, the number of years required is greater than four. To achieve transmission elimination for *Ascaris* and hookworm in 3–4 years, and within a 5–6 year time horizon, allowing for two further years of monitoring once MDA has stopped, requires coverage levels in both age groupings of around 80–90% (the highlighted numbers in the table). In many areas of endemic STH, LF is also present and the communities in these locations may have had a number of years of community-based MDA treatment (all age groupings). This makes life somewhat easier, as illustrated in Table 2 for *Ascaris* with a pristine R_0_ of 2.21. If two to 5 years of high community-based MDA coverage (for LF control) have taken place before the STH trial, then lower levels of coverage are required to break transmission in 2 to 3 years [28].

**Table 1:** Number of annual rounds in years to transmission elimination predicted by the deterministic model with fitted parameters to a medium transmission area for *Ascaris* (R_0_ = 2.34) and hookworm (R_0_ = 2.2). Albendazole drug efficacy is embedded in the calculations. Numbers in bold reflect predictions, based on the deterministic model, of crossing the transmission threshold within four years

Coverage of Pre-SAC & SAC (%)	Coverage of adults (%)						Coverage of adults (%)					
	0	20	40	60	80	100	0	20	40	60	80	100
0	na	na	na	na	na	na	na	na	14	7	**4**	**3**
20	na	na	na	na	na	na	na	na	11	6	**4**	**2**
40	na	na	na	20	15	13	na	na	10	5	**3**	**2**
60	16	11	9	8	7	6	na	na	9	5	**3**	**2**
80	7	6	5	5	**4**	**4**	na	na	8	5	**3**	**2**
100	5	**4**	**4**	**3**	**3**	**3**	na	na	8	**4**	**3**	**2**

**Table 2:** Number of annual rounds in years to transmission elimination predicted by the deterministic model with fitted parameters to a medium transmission area for *Ascaris* (R_0_ = 2.34) given 0, 2 or 5 years of community MDA for LF at an effective coverage level of 70% across each age grouping. Albendazole drug efficacy is embedded in the calculations. Numbers in bold reflect predictions, based on the deterministic model, of crossing the transmission threshold within four years

*Ascaris*: equilibrium							*Ascaris*: 2 years LF						*Ascaris*: 5 years LF					
Coverage of Pre-SAC & SAC (%)	Coverage of adults (%)						Coverage of adults (%)						Coverage of adults (%)					
	0	20	40	60	80	100	0	20	40	60	80	100	0	20	40	60	80	100
0	na	na	na	na	na	na	na	na	na	na	na	na	na	na	na	5	**3**	**3**
20	na	na	na	na	na	na	na	na	na	na	na	na	**1**	**1**	**1**	**1**	**1**	**1**
40	na	na	na	20	15	13	na	na	23	13	10	9	**1**	**1**	**1**	**1**	**1**	**1**
60	16	11	9	8	7	6	9	7	6	5	**4**	**4**	**1**	**1**	**1**	**1**	**1**	**1**
80	7	6	5	5	**4**	**4**	**4**	**4**	**3**	**3**	**3**	**3**	**1**	**1**	**1**	**1**	**1**	**1**
100	5	**4**	**4**	**3**	**3**	**3**	**3**	**2**	**2**	**2**	**2**	**2**	**1**	**1**	**1**	**1**	**1**	**1**

Note that for the trial arm in which only Pre-SAC and SAC are treated, no level of coverage for either *Ascaris* or hookworm is predicted to break transmission with 5 years unless past LF control over 5 years or so has reduced the value of R to just above unity.

Much parameter uncertainty surrounds key transmission processes of the major STH species. These include parasite aggregation as defined by the negative binomial parameter, k, the severity of density dependence in fecundity, adult worm life expectancy and the cross-transmission coefficients determining how infective stages released by one age group infect other age groups (33). Sensitivity analyses are required of defined ranges of parameter uncertainty to see how this influences the values in Tables 1 and 2. This is an essential step and it can also be performed in the stochastic simulations described later.

**Fig. 3:**
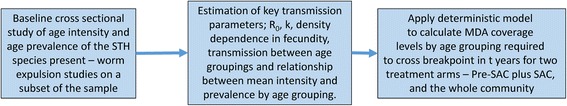
Flow chart summary of the steps in the suggested procedures and the epidemiological calculations required to arrive at the initial estimates of MDA coverage in each age grouping prior to performing the stochastic simulations of the trial

The steps in the suggested procedures, and the calculations required to arrive at these estimates of coverage, are summarised in Fig. 3. Following these initial calculations, the next stage involves deciding on a statistic to measure success or failure, and the simulation via an individual-based stochastic model of the full probability distribution of the time to breaking transmission, given achievement of the required drug coverage of the age groupings. This is addressed in the following section.

#### Statistic to measure success or failure, and which epidemiological variable to assess

The two outcomes of interest in the trial are transmission interruption and failure to interrupt transmission. In epidemiological terms this translates to elimination, with the prevalence and mean intensity falling to zero over a defined time period, and bounce back in both statistics to the levels prevailing before the trial started over a time scale largely influenced by the life expectancy of the adult worm [25]. The positive predictive (PPV) or negative (NPV) predictive value [36] are appropriate measures. The PPV and NPV are the proportion of positive and negative results in the trials (either measured by prevalence or mean intensity) that are true positive and true negative results, respectively. The NPV is defined as follows:

NPV = The number of true bounce backs detected/(The number of bounce backs detected (true negatives) + The number of eliminations wrongly detected as bounce backs (false negatives))

Conversely the PPV is defined as:

PPV = The number of true eliminations/(The number true eliminations detected (true positives) + The number of bounce backs wrongly detected as eliminations (false positives))

A small PPV indicates that most of the villages which are assessed to have eliminated transmission are false positives and vice-versa. A small NPV indicates that most of the villages that are assessed as having failed to eliminate transmission have indeed successfully interrupted transmission. In the event that past LF treatment coverage has already broken STH transmission, post cessation of the trial of STH treatment, the trajectories of prevalence and intensity will continue to decay.

**Fig. 4:**
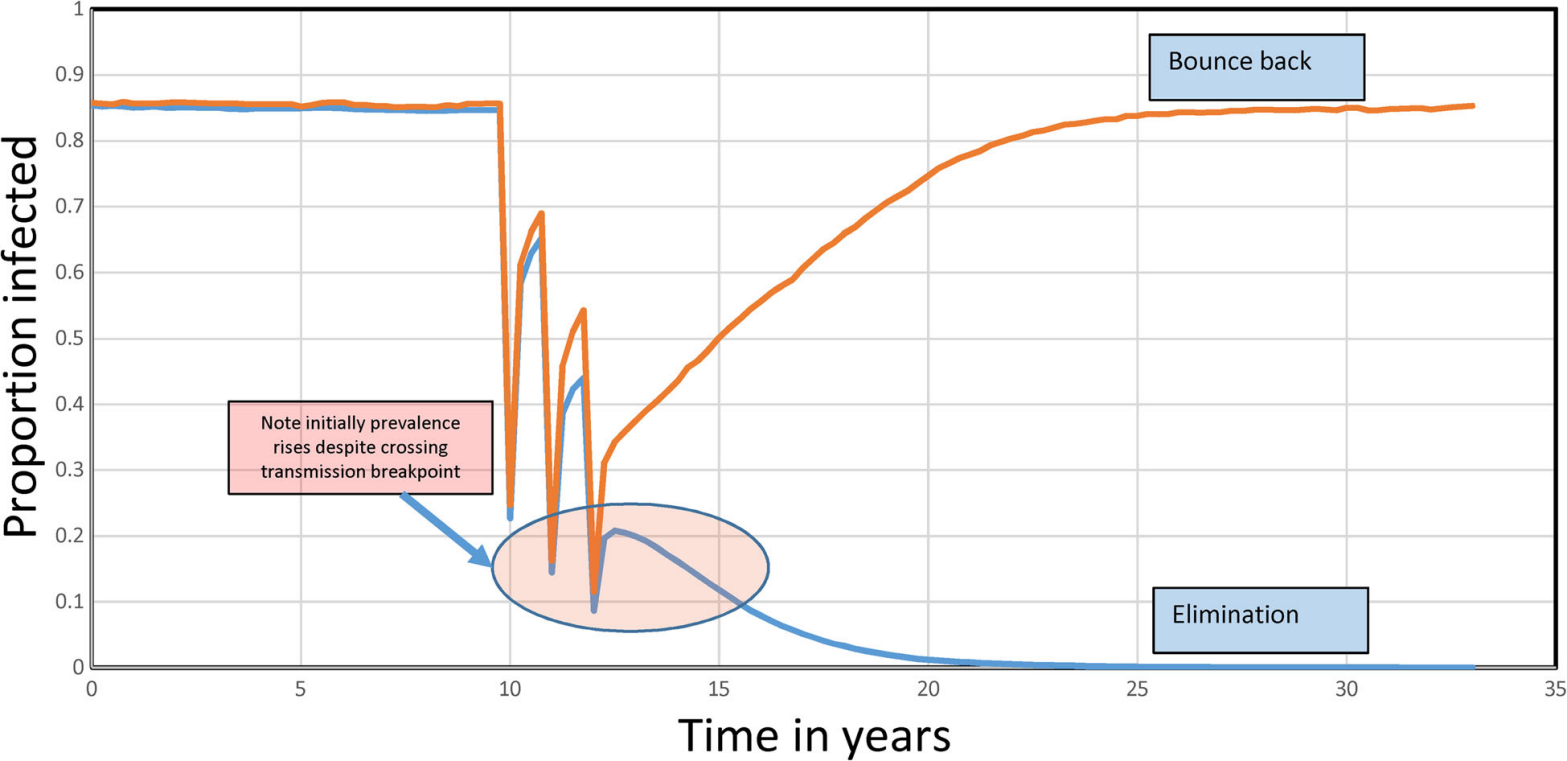
Stochastic simulations. Two stochastic simulations of three annual rounds of chemotherapy to control *Ascaris* (R_0_ = 2.2; k = 0.9, L = 1 year, ɣ = 0.07) (Infants 0% coverage, 90% coverage of Pre-SAC and SAC, 80% coverage of Adults) showing elimination and bounce back. The mean proportion infected (from 300 replicate runs) are shown for those simulations that result in elimination and those that result in bounce back (parameters as in Table 3)

The choice of these two statistics is governed by the fact that any trial to detect transmission elimination - say based on the prevalence of infection eventually falling to zero - will have to be run for some years post-cessation of MDA since the time trajectory of prevalence and intensity will be complex once the transmission threshold is crossed and extinction becomes the attractor in the non-linear dynamical system. This point is clearly illustrated in Fig. 4, where two replicate stochastic runs (see section below on stochastic models) of three annual runs of chemotherapy to control *Ascaris* infection resulting in either elimination or bounce back are recorded by changes in the prevalence of infection over time. Of all 300 replicates (representing different villages with 500 people per village) 88% of the runs resulted in elimination. However, note that when MDA ceases for a village in which elimination eventually occurs, the prevalence bounces upwards immediately after cessation before moving to zero over the following 10 years. This long timescale for elimination once the breakpoint is crossed is striking. However, note the different trajectories of the elimination run versus the bounce back run. In principle, it should be able to discriminate between these different outcomes 2 years after cessation of MDA. However, it is very clear that measuring either prevalence or intensity at the point of treatment cessation will fail to predict the eventual outcome.

Here the number in the definitions of PPV and NPV refers to the number of villages (as the base unit in the cluster randomized trial) in which either event occurs.

At low intensities of parasite transmission, detecting small changes in exposure to infection via a sensitive qPCR test is probably best based on the epidemiological measure of prevalence. This is the statistic used in the remainder of this paper. For a dioecious parasite, where two worms of the opposite sex must be within the host to produce viable offspring, the prevalence of infection could be defined as 1 minus the proportion of people with one or no worms. Given the uncertainties of past history with parasites with average life spans of 1 year or more, prevalence is defined as 1 minus the proportion of people with no worms at a particular point in time. Mean intensity could also be used to monitor the likelihood of elimination or bounce back.

#### Individual-based stochastic model of transmission and treatment

The first individual-based stochastic model of helminth transmission was published by Anderson & Medley [31]. The paper examined how various heterogeneities, such as predisposition to heavy or light infection, influenced the probability distribution of parasite numbers per host. More recently, Truscott and colleagues [35] have described an individual-based stochastic transmission model for STH to examine the impact of MDA. This structure is used in the results presented in this paper and the reader is referred to this publication for details of the event table and parameter assignments for *Ascaris*, *Trichuris* and hookworm based on published epidemiological studies that detail full cross-sectional surveys of prevalence and intensity of infection.

The approach adopted in the analyses is to employ the stochastic model to run replicate experiments in a defined number of villages. The number of villages, the average population size in each village, the dominant STH species and the intensity of transmission prior to the start of the trials are all parameters in the simulations. The time scale of the trials is set at 5 years with three annual rounds of chemotherapy. The demography of the villages is set as that of Kenya from the household survey 2003–2005. Migration in and out of the trial villages is set at zero in most of the experiments but how migration influences outcomes is examined in a separate set of experiments.

The deterministic model discussed in the previous section is employed to determine what level of coverage will lead to crossing the breakpoint in transmission with 3 years of annual treatment for a given STH species and initial R_0_ value.

Parameter uncertainty is also examined with respect to changes in R_0_, the negative binomial k value and the severity of density dependence on fecundity.

The focus is on the PPV, in terms of the percentage of villages detected as having eliminated transmission that in fact truly did eliminate infection. Sampling of each village population is not addressed, but in practice, as opposed to within the stochastic simulations where all infection levels in all people are known, this must be fully cross-sectional and adherence to treatment must be followed longitudinally.

## Results

### Simulation experiments

**Fig. 5:**
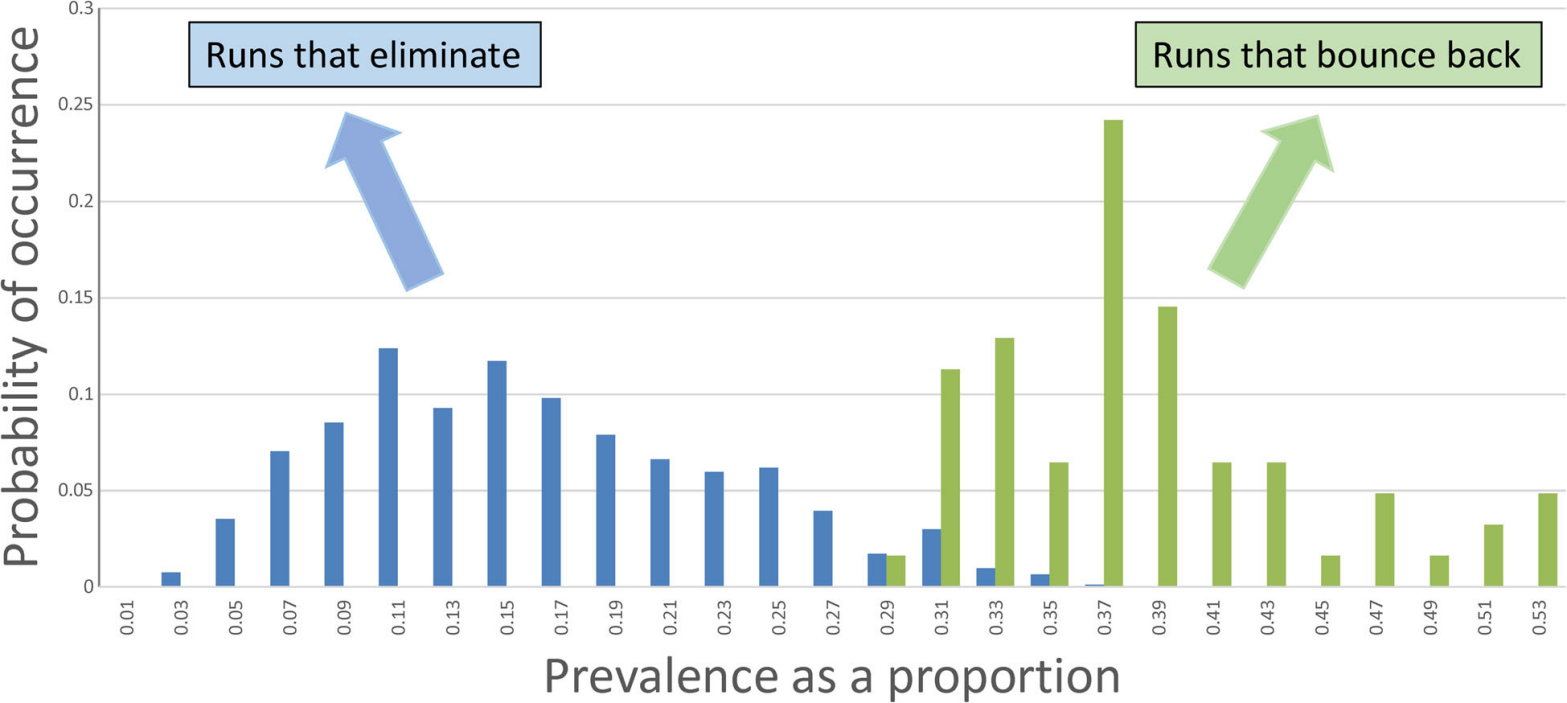
Prevalence of *Ascaris* after MDA. Illustrative example of the prevalence of *Ascaris* infection in a village at year 5 after three rounds of annual treatment measured followed by 2 years of no treatment. The graph records the frequency distribution of the proportion of a population of a village infected in villages in which transmission is eliminated (*blue* bars) and those in which bounce back occurs (*green* bars). In Figs. 6, 7, 8 and 9 all probabilities are conditional with the *blue* bars and *green* bars each summing to 1

The simulation experiments are all set to mimic a trial run over 5 years with a randomized set of villages of given population sizes with three annual rounds of MDA (with defined coverage levels in Pre-SAC, SAC and Adults) and 2 years of monitoring at the end of which the prevalence of infection in the total village is recorded (at time t = 5 years). In the replicated experiments, the frequency distributions of the number or proportion of villages with a prevalence of x (defined as a proportion infected) is recorded. An illustrative example for *Ascaris* is plotted in Fig. 5. If the trial criterion for elimination of transmission is a prevalence less than y at year 5, then the PPV and NPV can both be calculated from these frequency distributions created by the stochastic model.

In terms of a randomized cluster trial design, the villages would be chosen at random to fall into one of two groups (community wide treatment or Pre-SAC and SAC treatment only). The villages should be grouped by both the dominant STH species and the transmission intensity (low, medium or high) environments before randomization.

The critical issues here are what value should be assigned to the statistic y and what fraction of villages should be predicted to fall in the elimination category? The illustrative example portrayed in Fig. 5 provides a possible solution. For the first query, namely, the value of y, it should be a prevalence which does not permit too much overlap in the two distributions of elimination and bounce back. In the example shown this value of y is 0.27, or a prevalence of 27% at year two post-cessation of treatment. The second issue is the value of the predicted percentage of trials falling in the elimination category. The figure was 88% for the example shown in Fig. 5. Given the short duration of the community-based trial suggested (a total of 5 years), this figure should certainly be above 80% and preferably above 90%. It is to be hoped that if 5 years of LF treatment has occurred before the STH trial takes place, a much lower value of the statistic y could be used (say 5 to 15%) which will in turn influence the fraction of villages in which transmission ceases.

The next step is the calculation of the PPV and NPV. This is best illustrated by a series of examples of parameter assignments based on *Ascaris* infection detailed in a series of graphs of these distributions for various assumptions on village replicates and average population size per village.

**Fig. 6:**
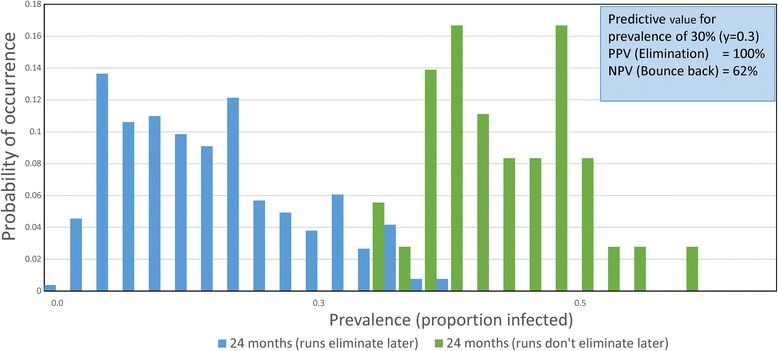
*Ascaris* prevalence distributions 1. Predicted distribution of *Ascaris* prevalences after three annual rounds of treatment and 2 years after the end of MDA for villages in which elimination occurred and villages in which bounce back resulted. The insert text box gives the PPV and NPV for a prevalence of 30%. These simulations were conducted for 300 villages each with a population of 500 people with MDA coverage of 0% infants - 90% pre-SAC - 90% SAC - 80% adults. Parameter values as defined in Table 3. The predicted percentage elimination in the replicates is 88%

Figure 6 records the first example, and plots the distribution of parasite prevalence after 3 annual rounds of treatment and 2 years after the end of MDA, for villages in which elimination occurred, and villages in which bounce back resulted. These simulations were conducted for 300 villages, each with a population of 500 people. The epidemiological parameter settings were as defined in Table 3 and derived from an epidemiological study in India [35, 38]. The levels of effective drug coverage for the community-based arm derived from the deterministic model to achieve elimination within 3 years were Pre-SAC and SAC = 90%, and Adults = 80%.

**Table 3:** Parameter values for the *Ascaris* stochastic simulations of MDA impact (from [33])

Parameter	Value
Basic reproductive number, R_0_	2.12
Density dependent fecundity parameter, ɣ	0.07
Parasite life expectancy in the human host, L	1 year
Negative binomial parameter, k	0.9
Mixing transmission parameter, β_ij_ (Pre-SAC; SAC; Adults)	0.22; 1.88; 0.53
Drug efficacy	0.99

**Fig. 7:**
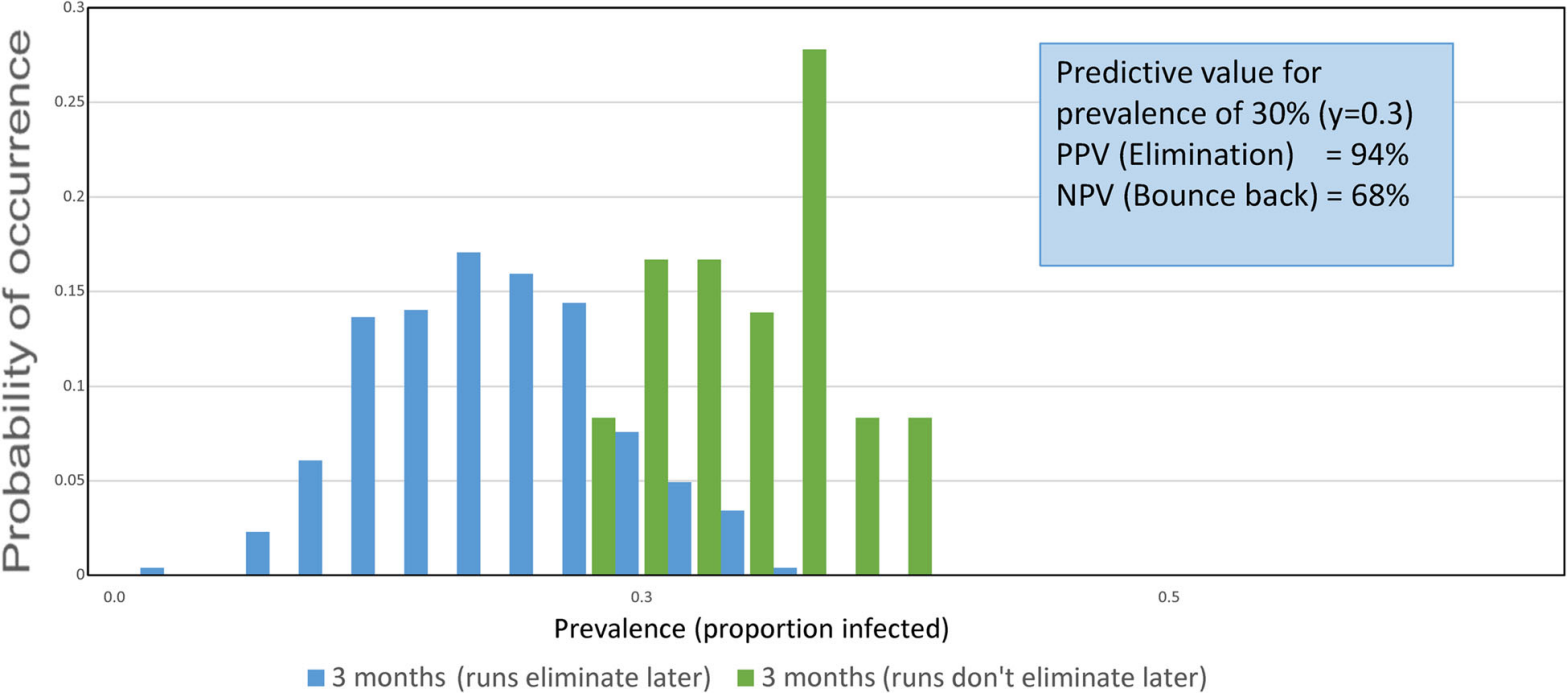
*Ascaris* prevalence distributions 2. Identical to Fig. 6 but with the observation period after the end of MDA set at 3 months and the prevalence threshold set at 30%

The insert text box in Fig. 6 gives the PPV = 100% and the NPV = 62% for a prevalence (the value of y) of 30% at the end of the 2 years of reinfection. The 95% confidence limits (98–100%) are recorded in Table 4 along with likelihood ratios and the raw data from the simulations summarised as the number of simulated village populations in which bounce back and transmission interruption were detected correctly (true) or incorrectly (false) [40, 41]. The overall percentage of villages in which transmission was interrupted was 88%, and the 95% confidence limits are again recorded in Table 4. If the point of observation, after the end of MDA, is decreased to 3 months, as opposed to 2 years, the chance of success in the detection of elimination falls a little as illustrated in Fig. 7, with PPV and NPV values of 94.4 and 67.7%, respectively, and wider confidence bounds (Table 5). Figure 7 shows overlapping distributions of prevalence in the elimination and bounce back villages at 3 months post-cessation of MDA This is a simple consequence of some infection continuing over the 3-month period in the egg-contaminated environment after transmission has been interrupted which acts to slightly blur the separation of those villages in which transmission has been interrupted and those in which bounce back will occur in the absence of any other changes in hygiene and sanitation. However, the difference is small, which may support an earlier assessment than at 2 years post-cessation of MDA.

**Table 4:** Confidence limits (95%) for PPV and NPV values for simulations presented in Fig. 6. Prevalence threshold 30% two years after cessation of MDA. Data from 300 village simulations with 500 people per village

Raw data from 300 simulations	Bounce back	Transmission interruption	Totals
Test positive	0	242	242
Test negative	36	22	58
Totals	36	264	300
		95% Confidence limits	
	Estimated value	Lower limit	Upper limit
Prevalence of interruption	0.880	0.836	0.913
Sensitivity	0.917	0.875	0.946
Specificity	1	0.880	1
Probability of simulation being either transmission interruption or bounce back			
Interruption	0.807	0.756	0.848
Bounce back	0.193	0.151	0.243
For predicted elimination - probability of being true or false			
True positive (PPV)	1	0.982	1
False positive	0	0	0.019
For predicted bounce back - probability of true or false			
True negative (NPV)	0.621	0.483	0.741
False negative	0.379	0.258	0.516
Likelihood ratios			
Positive (C)	Infinity	–	Infinity
Negative (C)	0.083	0.056	0.124
Positive (W)	Infinity	–	Infinity
Negative (W)	0.611	0.431	0.867

*Abbreviations*: *C* conventional, *W* weighted by prevalence

**Table 5:** Confidence limits (95%) for PPV and NPV values for simulations presented in Fig. 7. Prevalence threshold 30.0% three months after cessation of MDA. Data from 300 village simulations with 500 people per village

Raw data from 300 simulations	Bounce back	Transmission interruption	Totals
Test positive	15	254	269
Test negative	21	10	31
Totals	36	264	300
		95% Confidence limits	
	Estimated value	Lower limit	Upper limit
Prevalence of interruption	0.880	0.836	0.913
Sensitivity	0.992	0.929	0.980
Specificity	0.583	0.409	0.740
Probability of simulation being either transmission interruption or bounce back			
Interruption	0.9	0.855	0.930
Bounce back	0.1	0.072	0.145
For predicted elimination - probability of being true or false			
True positive (PPV)	0.944	0.908	0.967
False positive	0.055	0.032	0.092
For predicted bounce back - probability of true or false			
True negative (NPV)	0.677	0.485	0.826
False negative	0.322	0.173	0.514
Likelihood ratios			
Positive (C)	2.309	1.568	3.401
Negative (C)	0.064	0.034	0.124
Positive (W)	16.933	10.347	27.713
Negative (W)	0.476	0.277	0.817

*Abbreviations*: *C* conventional, *W* weighted by prevalence

**Fig. 8:**
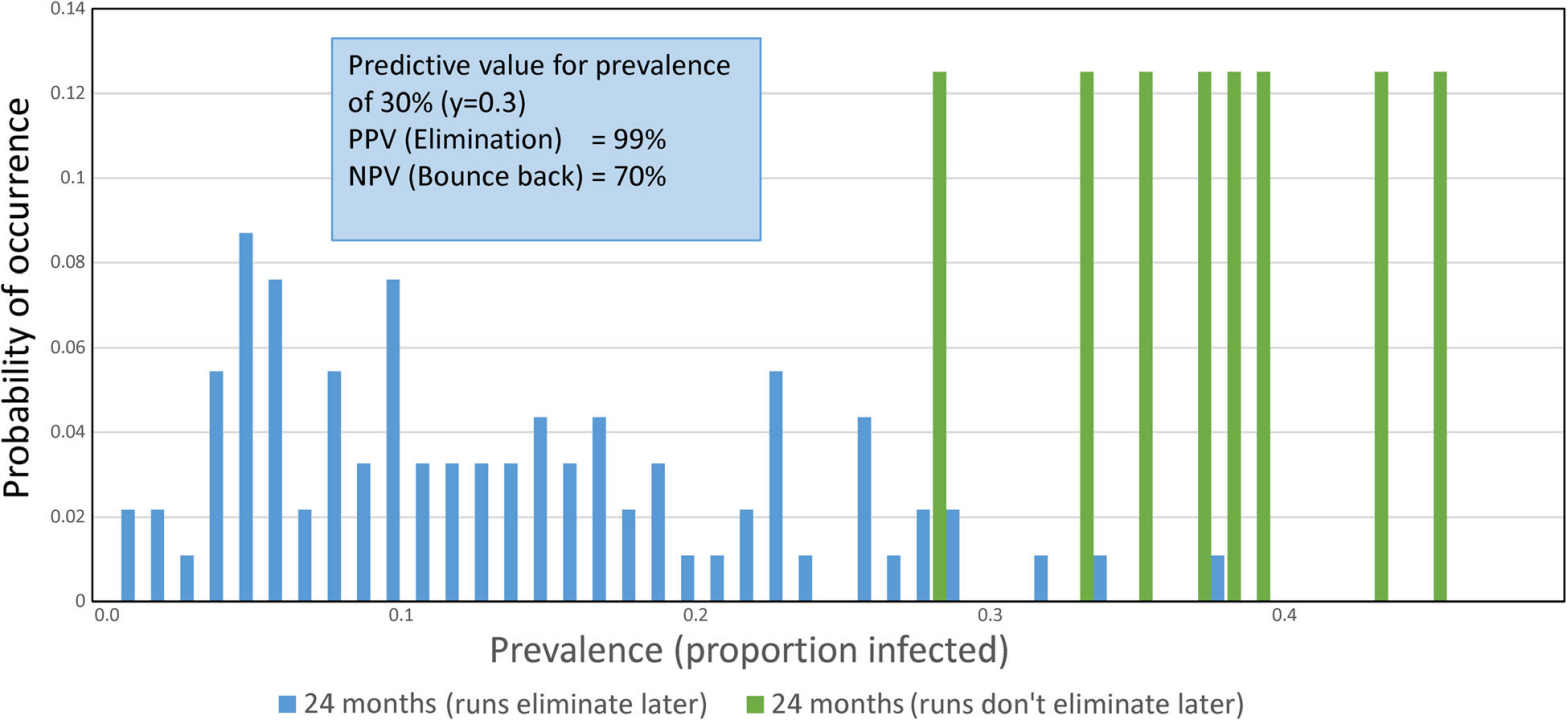
*Ascaris* prevalence distributions 3. Identical to Fig. 7 but with replication in 100 villages with 500 people per village. The percentage elimination is 92%

At this level of high replication (300 villages of 500 people per village), the chance of detecting elimination is good, with a very high PPV. If the number of villages is decreased to 100, each with the same population of 500, the calculated PPV and NPV values are reduced a little to 98.9 and 70%, respectively, but the percentage of villages in which elimination occurs is changed little (92%; confidence limits recorded in Table 6 and Fig. 8). There is little to be lost from this reduction of village replication, except in the NPV value detecting bounce back.

**Table 6:** Confidence limits (95%) for PPV and NPV values for simulations presented in Fig. 8. Prevalence threshold 30.0% two years after cessation of MDA. Data from 100 village simulations with 500 people per village

Raw data from 300 simulations	Bounce back	Transmission interruption	Totals
Test positive	1	89	90
Test negative	7	3	10
Totals	8	92	100
		95% Confidence limits	
	Estimated value	Lower limit	Upper limit
Prevalence of interruption	0.920	0.844	0.962
Sensitivity	0.967	0.901	0.991
Specificity	0.875	0.447	0.993
Probability of simulation being either transmission interruption or bounce back			
Interruption	0.9	0.819	0.948
Bounce back	0.1	0.052	0.180
For predicted elimination - probability of being true or false			
True positive (PPV)	0.989	0.930	0.999
False positive	0.011	0.001	0.069
For predicted bounce back - probability of true or false			
True negative (NPV)	0.700	0.354	0.919
False negative	0.300	0.081	0.646
Likelihood ratios			
Positive (C)	7.739	1.237	48.42
Negative (C)	0.037	0.012	0.117
Positive (W)	89.000	12.673	625.03
Negative (W)	0.428	0.153	1.201

*Abbreviations*: *C* conventional, *W* weighted by prevalence

**Fig. 9:**
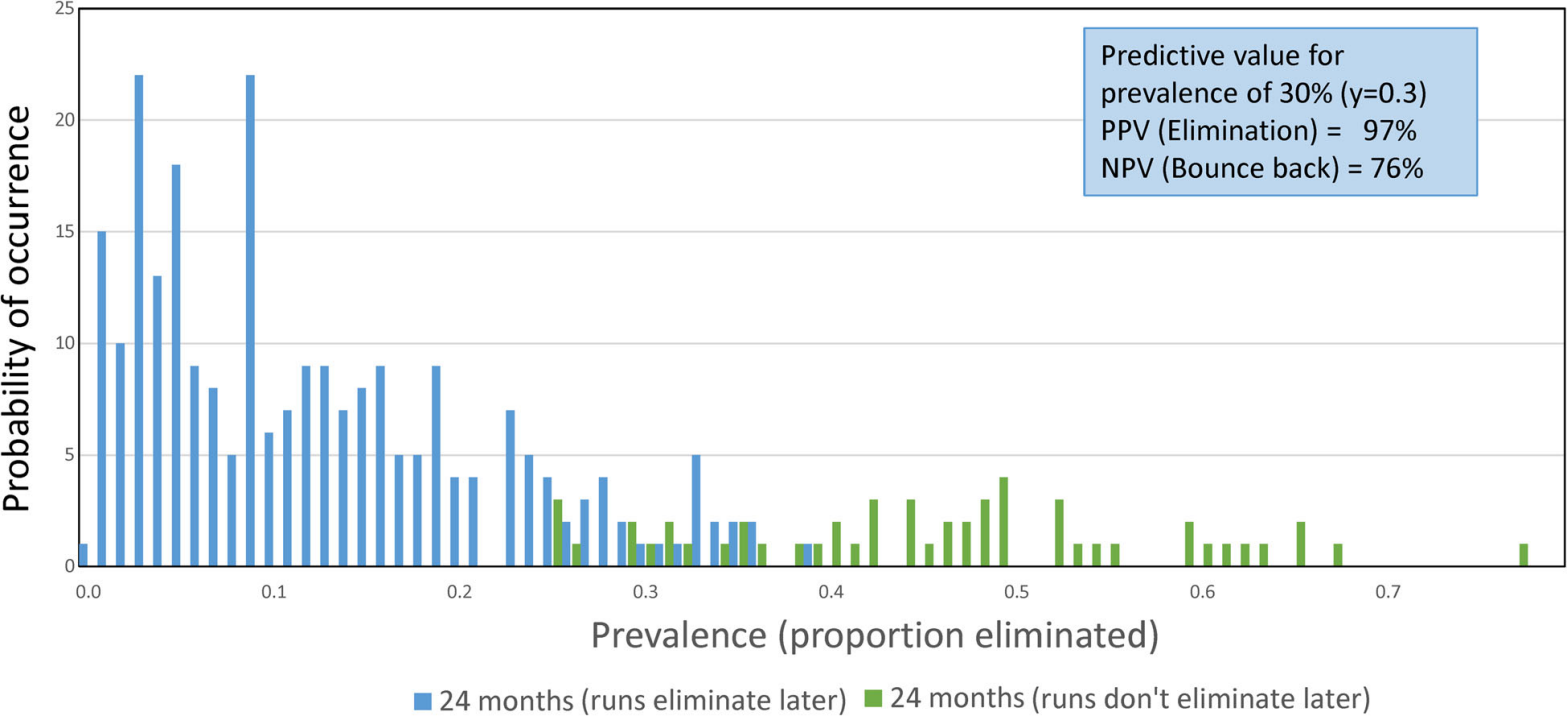
*Ascaris* prevalence distributions 4. Identical to Fig. 7 but with replication in 300 villages and 250 people per village. The percentage elimination is 82.0%

**Fig. 10:**
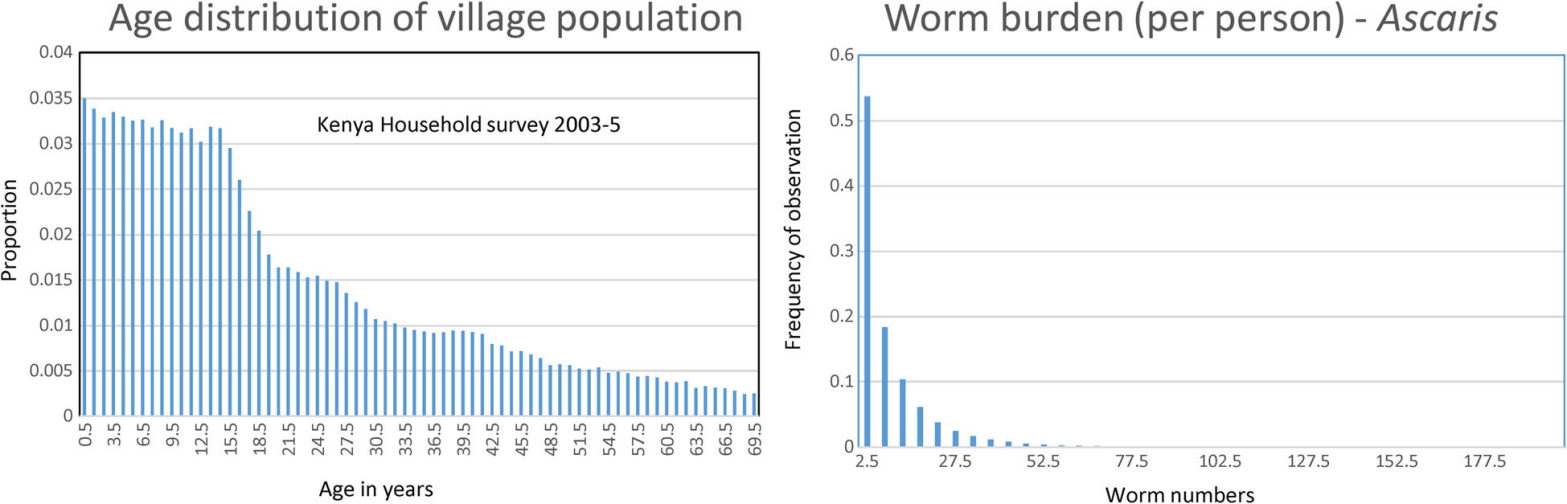
Effects of immigration. Immigration of infected people between village movements: basic data on demography and parasite distributions per person (k = 0.65, from [38])

Conversely, decreasing the number of people per village to 250, but keeping the number of village replicates in a cluster the same at 300, reduces the elimination percentage to 82%, and decreases the PPV to 97% and increases the NPV to 76% (Figs. 9 and 10 and Table 7). There is an obvious interplay between population size within a village and the number of villages assigned to a cluster within an arm of the trial in terms of the calculations of the PPV and NPV values plus associated confidence limits. Reducing population size within a village to around 250 people may be needed to match the reality of chosen village sites for the trial. It is encouraging that it is predicted that the percentage elimination remains at a high level for this population size (Table 7). In terms of the number of villages in each cluster, the simulations suggest 100 is adequate to keep the PPV value high with reasonable confidence limits (Table 6). In defined settings, logistical and resource constraints will be probably the greatest influence on the choice in this trade-off.

**Table 7:** Confidence limits (95%) for PPV and NPV values for simulations presented in Fig. 9. Prevalence threshold 30.0% two years after cessation of MDA. Data from 300 village simulations with 250 people per village

Raw data from 300 simulations	Bounce back	Transmission interruption	Totals
Test positive	6	232	238
Test negative	47	15	62
Totals	53	247	300
		95% Confidence limits	
	Estimated value	Lower limit	Upper limit
Prevalence of interruption	0.823	0.774	0.864
Sensitivity	0.939	0.900	0.964
Specificity	0.887	0.763	0.953
Probability of simulation being either transmission interruption or bounce back			
Interruption	0.792	0.742	0.837
Bounce back	0.207	0.163	0.258
For predicted elimination - probability of being true or false			
True positive (PPV)	0.974	0.943	0.990
False positive	0.025	0.010	0.057
For predicted bounce back - probability of true or false			
True negative (NPV)	0.758	0.630	0.854
False negative	0.242	0.146	0.5370
Likelihood ratios			
Positive (C)	8.300	3.903	17.640
Negative (C)	0.068	0.042	0.112
Positive (W)	38.667	17.544	85.220
Negative (W)	0.319	0.203	0.501

*Abbreviations*: *C* conventional, *W* weighted by prevalence

Once sites are selected, the simulations need to be conducted to look at the predicted percentage elimination and concomitantly, the PPV and NPV values for a series of defined prevalences after a defined period post the cessation of MDA. This period could be much shorter than 2 years (the time employed in the calculations displayed in Figs. 6, 8 and 9 and Tables 4, 6 and 7) as illustrated in Fig. 7 and Table 5.

### Adherence

It has already been mentioned that adherence to treatment at each of the three rounds of treatment is key, given the high coverage figures needed to break transmission in 3 years predicted by the deterministic model (80 to 90% of Pre-SAC and SAC and 80% of Adults).

**Fig. 11:**
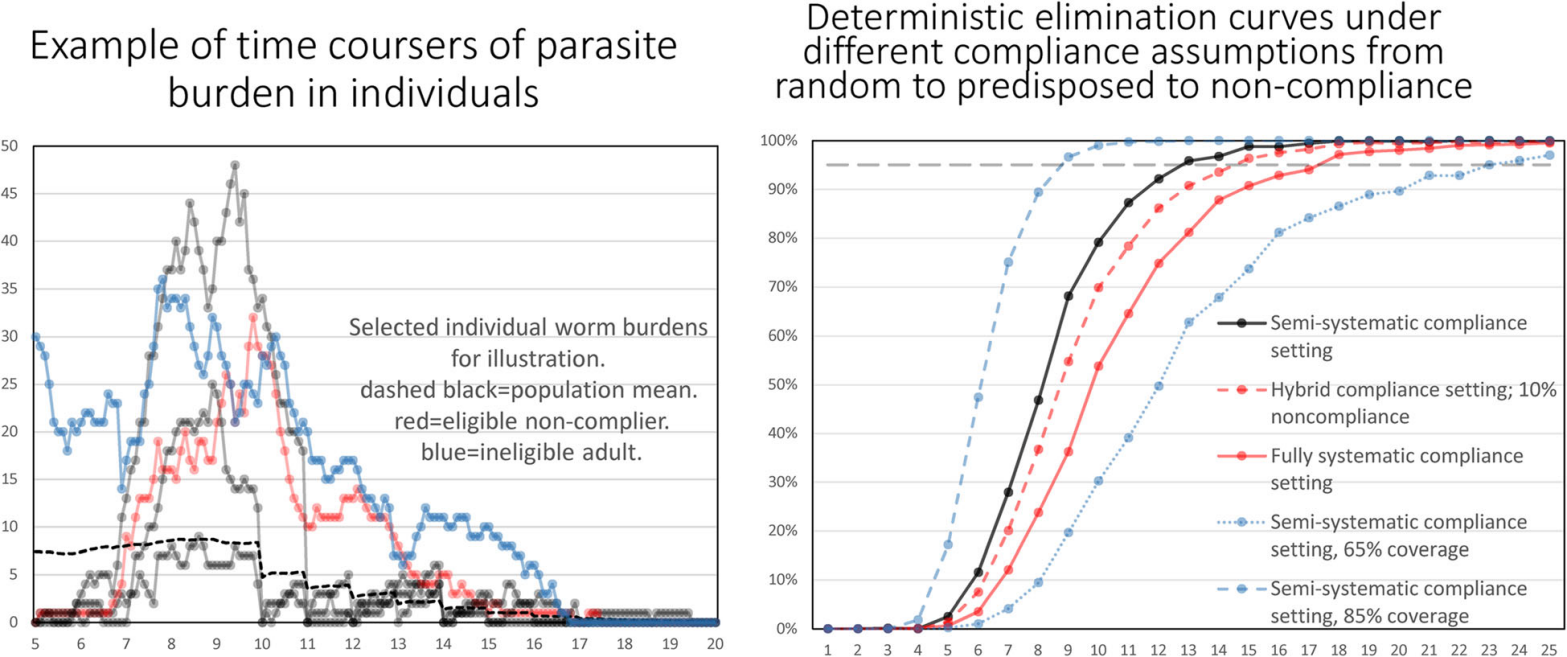
Adherence to treatment. The impact of various assumptions on adherence to drug treatment on the deterministic predictions the number of years of MDA at coverage levels of Pre-SAC and SAC at 90% and Adults at 80% before the breakpoint in transmission is crossed [39]

An analysis based on the model of the impact of various assumptions on adherence, from random at each round given a defined coverage level, to persistent non-adherers, shows that some patterns, in particular, persistent non-adherers at low to moderate levels, can significantly prolong the period of annual treatment required to break transmission beyond 3 years. This issue is illustrated in Fig. 11. These calculations highlight the importance of measuring, in a longitudinal observational programme, individual adherence at each round of annual treatment.

### Migration

**Fig. 12:**
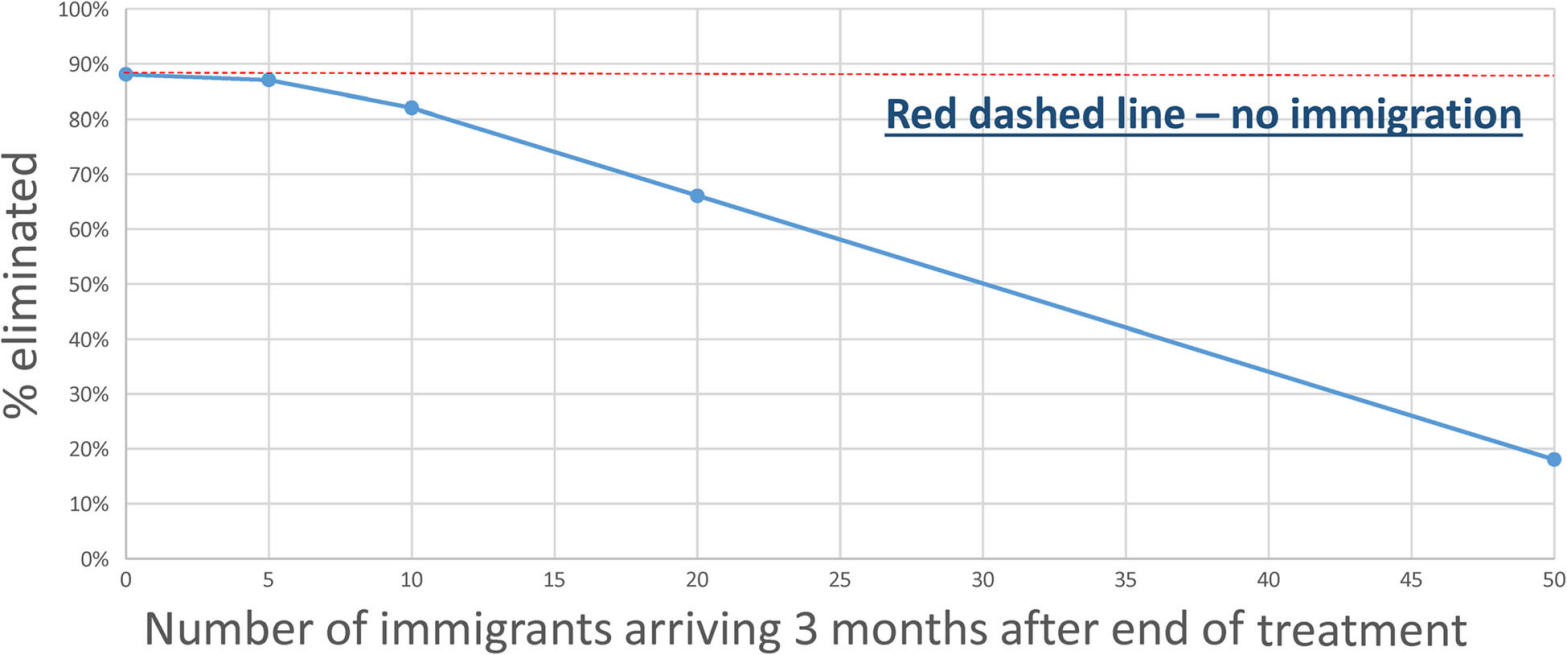
Effects of immigration. Immigration of people 3 months post end of 3 years of MDA - from outside the treated village - who are drawn at random from a worm distribution per person and human age distribution data identical to that pertaining in the village prior to treatment

**Fig. 13:**
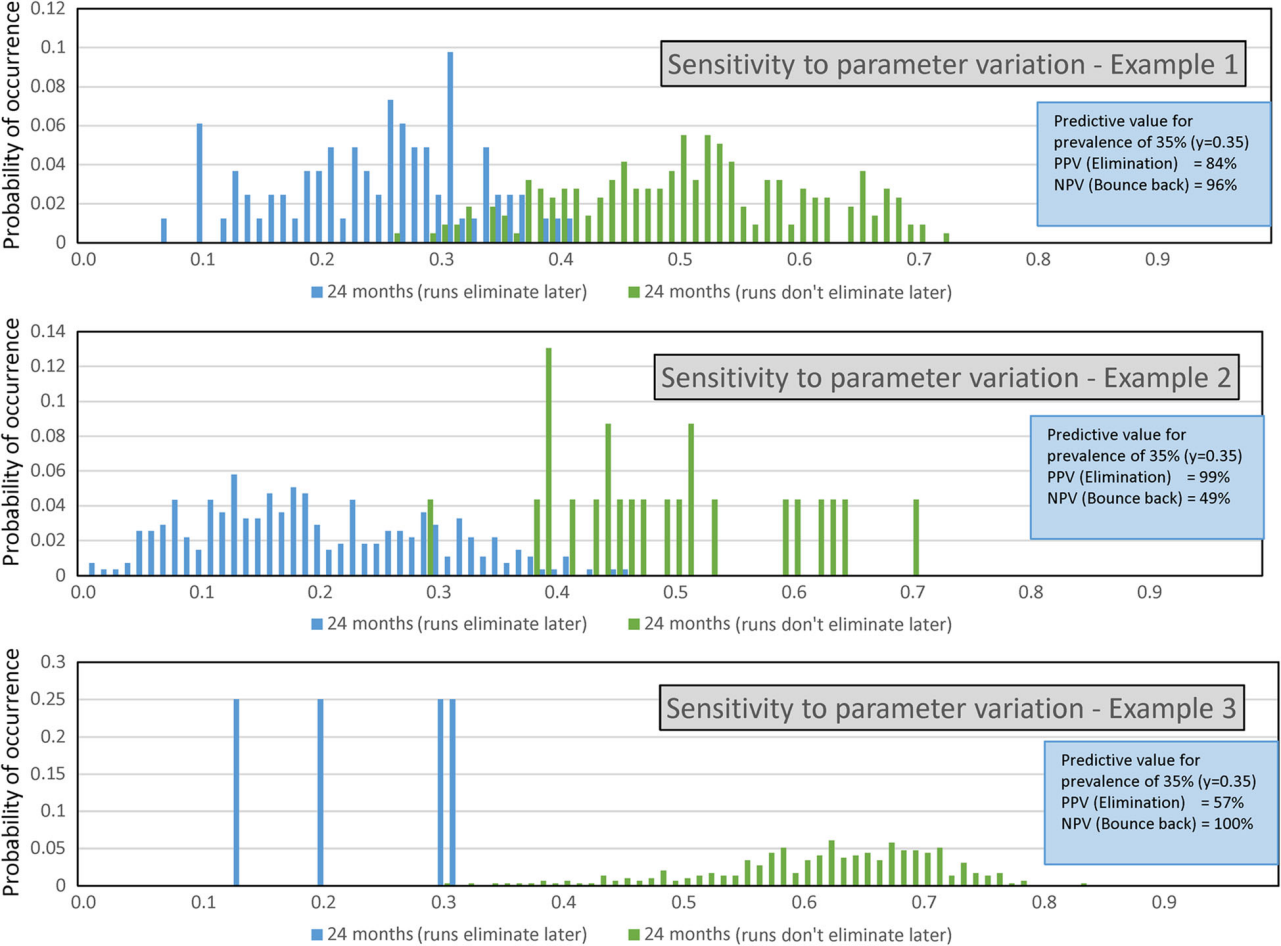
Parameter sensitivity. Some simulation experiments showing the sensitivity of the two distributions of elimination and bounce back 2 years after cessation of three annual rounds of chemotherapy to control *Ascaris* (MDA coverage as 0% infants - 90% pre-SAC - 90% SAC - 80% adults) with different parameters sets. Note several parameters vary simultaneously since the sets are taken from the Monte Carlo Markov Chain fitting of the model to age intensity data. Three different set of parameters chosen from one chain of an MCMC run are recorded. A stochastic model is used to convert epg counts for *Ascaris* to worm counts with density dependence in egg production built in

As noted earlier (Fig. 1), the adult male population (and in some cases females as well) in many poor communities, must migrate to towns or cities to find employment. These migrant labourers may return to their home villages at frequent intervals to visit family and friends. The impact of migration within any trial of breaking transmission can be examined using the stochastic model. The predictions of a simple simulation experiment are presented in Figs. 12 and 13. They are based, on the assumption of migrants being chosen first at random from the prevailing demographic age profile (illustrated in Fig. 12) and then ascribed a worm burden chosen at random from the overall population distribution prevailing in the village they migrated from (also illustrated in Fig. 12). The simulated outcomes of impact on the percentage elimination in a set of villages which migrants return to are recorded in Fig. 13 as a function of the number of migrants who return 3 months after the cessation of the three rounds of annual MDA. The calculations show clearly that high migration rates within villages of around 300 people can greatly reduce the chance of breaking transmission. The key issue is the measurement of migration rates, village by village, and season by season (migrating for employment is often seasonal). Treatment could be offered on the return of migrant labourers to their home village.

In the stochastic simulations portrayed in Figs. 5, 6, 7, 8 and 9, it was assumed that transmission between villages could be ignored. This is a key assumption, and needs to be verified by detailed analyses of migration and movement between villages, and also molecular genetic studies based on whole of partial genome sequencing studies of ‘who infects whom’.

### Sensitivity of stochastic predictions to parameter uncertainty

There are two major sources of parameter uncertainty. The first concerns individual parameter uncertainty due to difficulties in measurement. The second concerns variation in key parameters between the villages in a given cluster. Hopefully, the latter can be analysed once the baseline data is collected for each village and due account taken of such variation in the design of the trial. The former is more important in the context of this paper, and our analyses focus on how such variability influences the two distributions of elimination and bounce back. Figure 13 records three examples of parameter variation. The calculations were done by taking one chain of parameter estimates from a Monte Carlo Markov Chain (MCMC) calculation in fitting the model to data recording *Ascaris* infection intensity by age [33, 36]. Given the definition of R_0_ (with transmission and reproduction parameters in the numerator and mortality terms in the denominator), parameter values are correlated so sets were chosen from one fitting chain as the system converges to the best fit set.

**Fig. 14:**
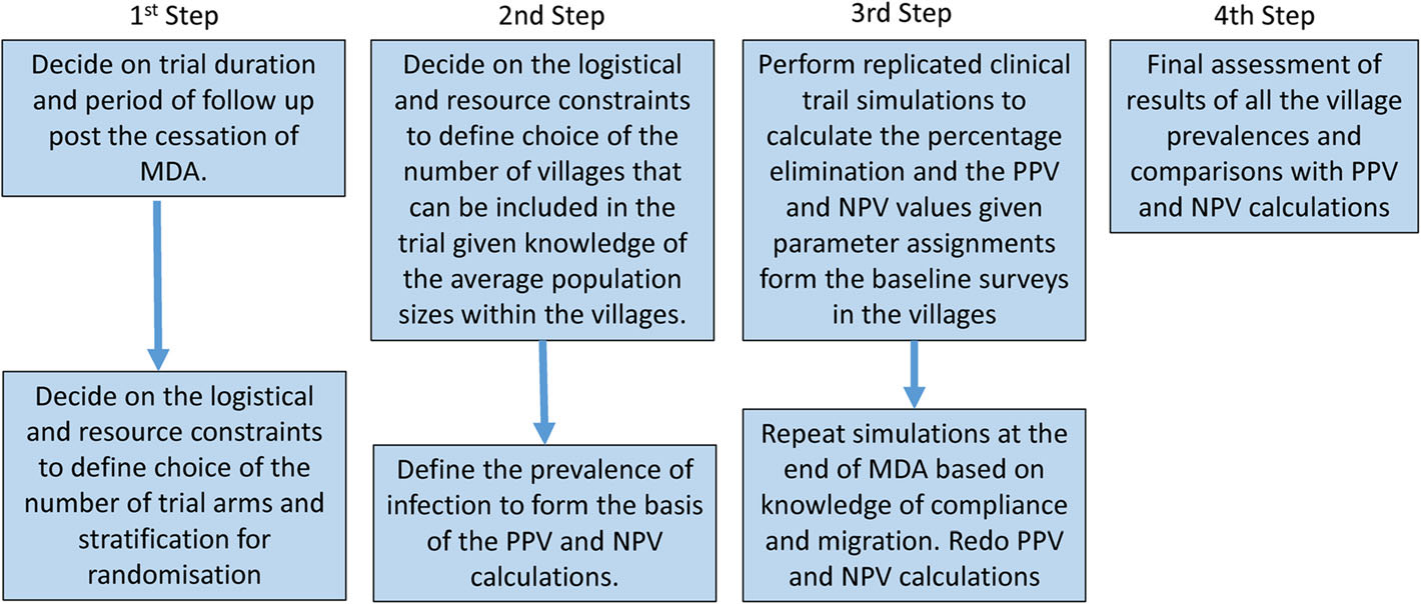
Flow chart summary of the steps (following on from Fig. 3) in the suggested procedures and the calculations required to arrive at the PPV and NPV values using the stochastic trial simulation model

Figure 14 shows clearly that parameter variation strongly influences the PPV and NPV values. The value of the overall transmission intensity is particularly influential. High values generate more bounce backs with a fixed level of coverage as to be expected. These results highlight the importance of parameter estimation at baseline in trial design.

## Discussion

The methodological approach described in this paper aims to help in the design of a randomized trial to detect transmission interruption of STH species under repeated rounds of MDA. Figures 3 and 14 outline what needs to be done in a series of steps (7 in total) to apply the stochastic randomized trial simulator to help design a ‘transmission interruption’ randomized cluster trial, with a number of stratifications; namely, which age groups to be treated (two arms), which is the dominant STH species (three possible stratifications) and which intensity of transmission setting (three possible stratifications). If LF-related MDA has taken place over the past 5 years before the start of the trial (a key selection criterion for DeWorm3 in site selection for the trials), most settings will have a low initial effective R value, but may be either low, medium of high R_0_ in terms of the pristine transmission potential in the chosen settings [28].

There are many uncertainties in some of these trial design steps, the most important of which is the resources that will be available to include sufficient villages in each cluster and over what duration of time the trials are to be run [29]. Others include average village population size, the accurate measurement of migration in and out of villages, the measurement of adherence to treatment, and how many arms/stratifications to include in the overall trial.

It may be necessary, due to logistical constraints and the resources available, to reduce the number of stratifications. An obvious simplification is the treatment of Pre-SAC and SAC only - since this will fail in most settings. However, one desired output from the trials is to influence WHO policy, which at present specifies the treatment of these two groups. If that arm cannot be discarded, the other stratification options are to focus on just one STH species such as hookworm. This parasite is somewhat easier to eliminate than say *Ascaris* (because of its typically rapid bounce back time due to the 1 year average lifespan in the human host) or *Trichuris* (two drugs needed to achieve good efficacy) because of its slower dynamic population turnover relative to the other two major species. A further option is to only conduct trials in low R_0_ transmission settings (or after many rounds of LF treatment such that the effective R is low).

Other issues concern the setting of the prevalence level, 2 years after MDA cessation, at which to estimate the PPV and NPV values - and what levels of these two statistics should be aimed for [29]? A sensible value for prevalence, to insure high PPV values, will have to be selected for each trial site and perhaps for each village based on simulation calculations. The selection criteria should be high PPV and NPV values and tight 95% confidence limits. This should allow good discrimination between the probability distributions for the elimination and bounce back outcomes. If transmission is very low prior to the start of the trial this could be set at 5%. Population size within villages needs to be sufficient not to exacerbate chance or stochastic effects. The trial simulations suggest a lower bound of 250 people per village is adequate to give high PPV and NPV values‚ if a reasonably high fraction of the villagers are sampled at the end of MDA. Population extinction by chance alone is more likely in small populations of hosts‚ hence robust conclusions that can be applied broadly should be based on large community sizes in the range of 200 to 300 people per village. How many villages to include in a cluster is also an important issue, which ultimately will be controlled by the resources available, and logistical and technical issues within the countries chosen for the trials. However, the number of villages in each cluster will need to be calculated, given the need to compare the probability distributions for the two outcomes and assess the degree of overlap. There is an obvious trade-off between the magnitude of the PPV and NPV values. Which to maximise in sample calculations will depend on which is the more important outcome to detect accurately, elimination or bounce back? In this trial it would seem that maximising the likelihood of correctly detecting elimination of transmission is a higher priority. These issues are discussed in more detail in a companion paper [29].

Perhaps the main challenges lie more with the acquisition of good quantitative epidemiological data at the baseline on infection, adherence to drug treatment and migration. STH epidemiology has not advanced rapidly over the past few decades in terms of applying modern statistical tools to parameter estimation and good surveys practices both horizontal and longitudinal. The small numbers of estimates of the degree of parasite aggregation, the presence of predisposition and the basic reproductive number, R_0_, in the published literature are a reflection of this past trend. The importance of this relates to the degree of variation present between villages within a cluster. This must be measured carefully, as well illustrated in the section on parameter uncertainty (Fig. 14). The variability in a cluster has two sources, true uncertainty in measurement and variation in transmission intensity and other population dynamic parameters between the villages. Diagnostics will clearly play an important role in the validity of a chosen epidemiological measure for transmission interruption. There is a major difference between Kato Katz and q-PCR measures (see Fig. 2). It is to be hoped that the conduct of the DeWorm3 trials will stimulate greater quantitative precision in STH epidemiological study.

The problem with the choice of the PPV/NPV statistics is in their dependency on the chosen prevalence at a defined time point post cessation of MDA at which to perform the PPV/NPV calculation. However, confidence limits and sensitivity and specificity, can be defined for these two statistics in relation to the chosen prevalence value. The aim is to keep PPV and NPV values high with tight 95% confidence bounds. As noted earlier, it may be best to focus on keeping the PPV value high in the choice of sample size of people per village and the number of villages per cluster.

The calculations presented in this paper are aimed to stimulate discussion on trial design before protocols are set for a series of chosen study sites. They are also aimed to promote the use of individual-based stochastic models of non-linear parasite transmission, firmly grounded on parameters estimated from field study sites, to sharpen trial design for infectious disease control assessments and hence to reduce the likelihood of errors in design and associated costs. Where significant non-linearities are present in dynamical systems, intuition alone is not a good guide to the interpretation of observed pattern. Such non-linear patterns are part of the life-cycles of all helminth infections of humans.

One of the major publications of the pioneering helminth epidemiologist Nelson Hairston begins with a quote from L. W. Hackett, taken from the book entitled ‘Malaria in Europe’ published in 1937 [37]. It is well worth repeating, in light of the complexities introduced in epidemiological study by the non-linear transmission dynamics of helminth parasites. “A closer collaboration between biometricians and parasitologists, and a better acquaintance ship of each with the methods of the other, is one of the most useful things we can work for today”. This sentiment is as applicable in 2016 as it was in 1937 when Hackett’s book was first published.

## Conclusions

A stochastic individual based model is developed to mimic the transmission dynamics of soil transmitted helminths (STH) and their control by mass drug administration (MDA). The model is employed to mimic cluster randomized trials of the impact of MDA. The calculations are designed to consider various trial arms and stratifications; namely, community-based treatment and pre-school aged children (Pre-SAC) and school aged children (SAC) only treatment (the two arms of the trial), different STH transmission settings of low, medium and high, and different STH species mixes. The complications introduced by the choice of statistic to define success or failure, varying adherence to treatment, migration and parameter uncertainty are analysed. The simulations demonstrate the power of the methods in helping to design cluster randomized trials for infectious disease agents with non-linear transmission dynamics.
